# Laterally Positioned Flap with Subepithelial Connective Tissue Graft Modified One-Stage Procedure for the Treatment of Deep Isolated Gingival Recessions in Mandibular Incisors

**DOI:** 10.1155/2021/2326152

**Published:** 2021-08-05

**Authors:** Alvaro Francisco Bosco, Juliano Milanezi de Almeida, Belén Retamal-Valdes, Renata Tavares, Jessica M. Latimer, Diego Messina, Matheus Henke, Laura Gantes Rodrigues Dias, Thiago Marchi Martins

**Affiliations:** ^1^Department of Surgery and Integrated Clinic, School of Dentistry, São Paulo State University, Araçatuba, São Paulo, Brazil; ^2^Department of Periodontology, Dental Research Division, Guarulhos University, Guarulhos, São Paulo, Brazil; ^3^Department of Oral Medicine, Infection, and Immunity, Harvard School of Dental Medicine, Boston, MA, USA; ^4^Department of Semiology and Clinic, Periodontology Discipline, Federal University of Pelotas, Pelotas, Rio Grande do Sul, Brazil

## Abstract

The laterally positioned flap (LPF) has been proposed as a promising treatment for isolated gingival recessions (GRs) in mandibular incisors. Several modifications have been proposed to reduce the risk of gingival recession (GR) at the donor tooth site. Therefore, the aim of this was to describe a modified one-stage procedure of performing the LPF associated with the subepithelial connective tissue graft (LPF + SCTG) with the modifications for the treatment of deep isolated GR in mandibular incisors. The modified one-stage technique (LPF + SCTG) is unique because it was presented being bilaminar with tunneled connective tissue graft (CTG) in the adjacent tooth and extended to the flap donor site, without a submarginal incision in the adjacent tooth, taking the entire band of the keratinized tissue (KT) into the flap. In addition, 3 clinical cases were described using this surgical technique. Three healthy patients with Cairo RT1 or RT2 GRs on teeth 31 or 41 were treated with the LPF + SCTG technique. Probing depth (PD), clinical attachment level (CAL), complete root coverage (CRC), mean root coverage (MRC), recession depth (RD), and keratinized tissue width (KTW) were assessed at baseline and in the follow-up periods of 18, 24, and 48 months, in the cases 1, 2, and 3, respectively. The LPF + SCTG with the modifications presented is a predictable approach for the treatment of deep isolated RT1 and RT2 GRs in mandibular incisors that are well positioned in the bone envelope with the presence of KTW adjacent to GR and adequate vestibule depth in the donor area of the flap.

## 1. Introduction

Gingival recession (GR) is the apical shift of the gingival margin with respect to the cementoenamel junction (CEJ), with exposure of the root surface to the oral environment [1]. This condition may promote the occurrence of dental hypersensitivity and root alterations in the cervical area. In addition, it may become one predisposing factor for plaque accumulation [2] or may be considered an esthetic problem by the patient [1]. The overall prevalence of GR in the adult population has been reported to be > 60% [3], while the central and lateral mandibular incisors were the most frequently affected teeth [4, 5].

Coronally advanced flap (CAF) with subepithelial connective tissue graft (SCTG) is now considered the gold standard procedure for the treatment of GR [6–8]. Due to unfavorable local anatomical conditions, the CAF has been contraindicated in some clinical situations [9]. Thus, the clinician should take into consideration the soft tissues located laterally to the recession defect into consideration to evaluate the possibility of performing the laterally positioned flap (LPF) [9, 10].

Numerous modifications to the original laterally sliding flap proposed by Grupe and Warren [11] have been published in order to reduce the risk of GR at the donor site [12, 13]. Bosco et al. [14] proposed an internal bevel performed in the graft recipient area. This modification in technique promotes better flap adaptation to the recipient area and favors the esthetic result by reducing visibility of the incision line perception after scarring. Zucchelli et al. [9] suggested the coronal advancement of the laterally moved flap and a different thickness during flap elevation with a submarginal incision in the adjacent tooth. Recently, other authors have proposed the association of an SCTG coverage by LPF without a submarginal incision in the adjacent tooth, taking the entire band of the keratinized tissue (KT) into the flap [15]. More predictable prognosis was associated with gingival thickness (GT) of 0.8–1.2 mm [16] and a thickness < 1 mm that could affect the percentage of complete root coverage (CRC) [17]. In this context, the modified one-stage technique (LPF + SCTG) is unique because it was presented being bilaminar [18], with tunneled CTG in the adjacent tooth and extended to flap donor site, without a submarginal incision in the adjacent tooth, taking the entire band of the keratinized tissue (KT) into the flap and the inversion of bevels in relation to Parkinson et al. [13]. Therefore, the aim of this manuscript was to describe a modified one-stage procedure of performing the laterally positioned flap with subepithelial connective tissue graft (LPF + SCTG) with the modifications for the treatment of deep isolated GR in mandibular incisors.

## 2. Case Description

### 2.1. Surgical Technique (LPF + SCTG Modified-Figure 1)

#### 2.1.1. Clinical Indications and Contraindications

“This technique is a time-efficient, less invasive (one-stage procedure), and highly esthetic treatment option for managing isolated Cairo RT1 and RT2 GRs, in teeth well positioned in the bone envelope or slightly buccal where odontoplasty is possible, mainly in the mandible in the presence of a thin gingival phenotype and little or no band of KT, apical in the recession defect, with the presence of KT adjacent to GR (minimum of 2 mm), and adequate vestibule depth in the donor area of the flap. Instead, in the Cairo RT3 GRs, this technique would not be indicated due to the presence of extensive interproximal CAL and lack of predictability for root coverage”.

After the full-mouth periodontal examination, all patients receive a dental prophylaxis including oral hygiene instruction (OHI), supragingival scaling, and professional tooth cleaning. For teeth with GR, a coronally directed roll tooth brushing technique is recommended to minimize toothbrushing trauma to the gingival margin. On the day of surgery, patients rinse with 15 ml of 0.12% chlorhexidine gluconate mouth rinse for 1 minute. In addition, anti-inflammatory medication (600 mg ibuprofen), 1 hour before the surgical procedure, is indicated.

Local anesthesia of the bilateral mental and lingual nerves is achieved with 2% mepivacaine (MEPIADRE 100, DFL). Only the portion of the exposed root with attachment loss should be instrumented with curettes. The roots are conditioned for two minutes with cotton pellets soaked with 100 mg/ml tetracycline hydrochloride solution, followed by abundant irrigation with saline solution [15].

**Figure 1:**
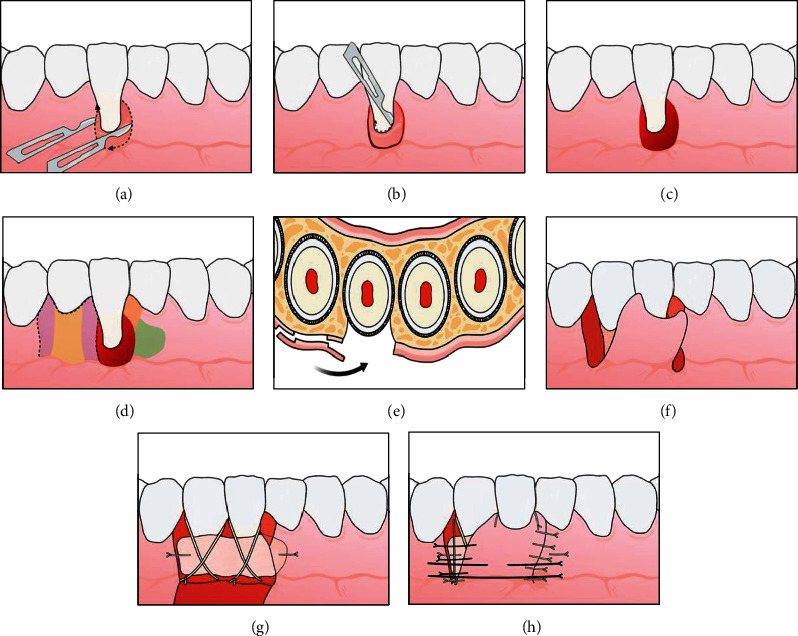
Schematic representation of the surgical technique. (a) Internal and external bevel incision, whereas flap will be positioned from tooth 42 to 41. (b) Intrasulcular incision in tooth 41. (c) Deepithelialization area providing laterally positioned flap (LPF) with an anchorage bed. (d) Delimitation of mixed thickness flap (purple color: split thickness; yellow color: full thickness) and preparation of surgical bed to receive connective tissue graft (CTG) (orange color: papilla deepithelialized; green color: tunnel). (e) Cross-section showing different thicknesses of flap that will be positioned laterally. (f) Flap prepared and positioned laterally. Papilla mesial of tooth 41 deepithelialized (orange color in (d)). (g) CTG positioned and stabilized in the created tunnel (green color in the (d)), over the gingival recession (GR) defect with extension to the flap donor area. (h) Flap positioned and stabilized with suture.

The surgical technique begins at the level of CEJ with an internal bevel incision using a 15C blade (2 to 3 mm from root surface) extending in the direction of the alveolar mucosa, bypassing the apical portion of the GR defect continuing with an external bevel incision along the distal gingival margin (1 mm from root surface) ending at the dental surface at the level of the CEJ [14, 15] (modification that was made to the original one [13]) (Figure 1(a)). After an intrasulcular incision (Figure 1(b)), this area is deepithelialized with the use of a 15C blade kept parallel to the external gingival surface. Connective tissue area lateral and apical to the root exposure provides the LPF with an anchorage bed (Figure 1(c)).

In the tooth adjacent to the GR, an intrasulcular incision is performed in the horizontal direction in order to contour the mesial and distal papillae (modification that was made to the original one [9]), continuing with a vertical beveled incision that starts at the level of the base of the papilla on the mesiobuccal surface of the second or third tooth adjacent to GR, extending into the alveolar mucosa [15]. The flap of mixed thickness is elevated, with split thickness in the papillae (Figure 1(d)-purple color) and full thickness (Figure 1(d)-yellow color) in the center up to the limit of the mucogingival junction and long enough to cover the mesial-distal extension of the avascular root area of the GR defect (Figures 1(d) and 1(e)). Once the mucogingival line is reached, apical split-thickness flap elevation is continued, keeping the blade parallel to the bone surface. Flap elevation is terminated when it is possible to passively move the flap laterally above the exposed root and reach a level coronal to the CEJ (Figure 1(f)).The papillae of the recipient site (marginal area of prepared tunnel) is carefully deepithelialized with a 15c blade (Figure 1(d)-orange color). The CTG is harvested from the hard palate in the region between the maxillary first premolar and the second molar by de-epithelialized free gingival graft (D-FGG) [19] technique. The donor area of the palate received protection from the epithelialized portion of the graft stabilized by sutures [19]. Appropriately sized CTG is positioned mesially, inside the tunnel prepared, (Figure 1(d)-green color) (modification that were made to the original one [14]) with the remaining portion covering the defect area and the distally adjacent tooth (or teeth) at the level of CEJ. CTG must be stabilized with two simple interrupted sutures (absorbable thread 5.0), one on the tunnel prepared and one on the papilla opposite to the defect site (Figure 1(g)). Subsequently, the flap will then be laterally positioned and secured with suspensory sutures (monofilamentar thread 5.0) and simple interrupted sutures to approximate margins and secure the LPF margin in the recipient site (Figure 1(h)).

Patients receive strict postoperative instructions, including medication (600 mg ibuprofen, every 12 hours). They are also instructed not to brush their teeth in the treated area, but to rinse with 15 ml of 0.12% chlorhexidine gluconate solution, twice a day for 1 minute for 14 days. Sutures at the donor site are removed 7 days postoperatively, and sutures in the grafted site are removed 2 weeks postoperatively. The patients are again reinstructed with regard to mechanical plaque control of the treated area and must continue with the chlorhexidine rinse instructions for 14 additional days.

All patients were informed about the treatment plan, the surgical technique, postoperative recommendations, and possible complications, and informed consent was obtained from the patients.

### 2.2. Case 1

**Figure 2:**
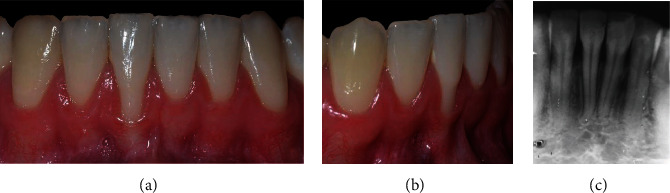
Case 1. (a) Frontal view, gingival recession of 4 mm high by 3 mm wide. (b) Right lateral view. (c) Periapical radiograph, 2 mm radiographic interproximal bone loss mesial and distal.

The patient, a 22-year-old Caucasian man, nonsmoker, systemically healthy, presented with the main complaint of difficulty with cleaning the region of tooth #41 and fear of losing the tooth, at the Department of Periodontology, Federal University of Pelotas (Pelotas, Rio Grande do Sul, Brazil), in April 2018. The patient had previously undergone orthodontic treatment. According to the clinical and radiographic examination, a RT2 RG was observed on the vestibular surface of the right mandibular central incisor (4 mm high and 3 mm wide at the height of the CEJ), with a probing depth (PD) of 3 mm in the proximal and 2 mm of 2 mm measured on the on free surfaces, clinical attachment level (CAL) of 6 mm on the buccal surface, radiographic interproximal bone loss mesial and distal sites, slight buccal prominence (dental linguoversion), slight dental extrusion, biofilm accumulation, protruding occlusal interference, and thin periodontal phenotype (Figure 2).

**Figure 3:**
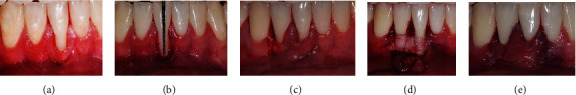
Case 1. (a) Vertical internal bevel incision extending into alveolar mucosa and external bevel incision along distal gingival margin of the recession defect and extending into alveolar mucosa. (b) Deepithelialized area after intrasulcular incision. (c) Flap of mix thickness. (d) Connective tissue graft positioned and stabilized. (e) Laterally positioned flap without tension and secured with sutures on recipient site.

After supragingival periodontal therapy, the protrusive occlusal interference in #41 tooth was subsequently adjusted to balance the contacts and improve the distribution of forces. To adjust this, occlusal contact markings were made at maximum habitual intercuspation, protrusion, and right and left laterality. For selective wear, spherical and tapered diamond (KG Sorensen, Cotia, São Paulo, SP, Brazil) tips were used, in addition to abrasive rubber, discs, and polishing paste (TDV Dental, Pomerode SC, Brazil), until the establishment of balanced contacts during mandibular movements were established, in order to maintain a mutually protected occlusion. Two weeks after the occlusal adjustment, periodontal surgical treatment was performed using the LPF + SCTG technique (T.M.M.), as previously described (Figure 3).

The CTG, with an average thickness of 1 mm, was removed from the palate, in the region between the first maxillary premolar and the first molar, using the D-FGG technique [19]. The CTG extended high enough into the mesiodistal direction, to cover teeth 41 and 42, high to cover the exposed root surface and remain within the tunnel created under the initial mesial incision of 41 tooth, in order to be covered by LPF later (Figure 3).

### 2.3. Case 2

**Figure 4:**
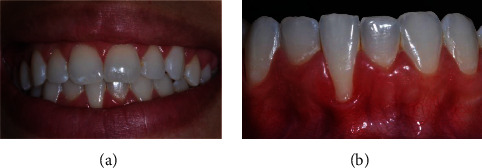
Case 2. (a) Frontal aspect of smile. (b) Frontal view, gingival recession of 4 mm high by 3 mm wide.

The patient, a 21-year-old Caucasian woman, nonsmoker, systemically healthy, presented with main complaint of difficulty with cleaning the region of tooth #41 and esthetic change when smiling, at the Department of Periodontology, Federal University of Pelotas (Pelotas, Rio Grande do Sul, Brazil). According to the clinical and radiographic examination, a RT1 RG was observed on the buccal surface of the right mandibular central incisor (4 mm high and 3 mm wide at the height of the CEJ), with a PD of 2 mm in the proximal and 1 mm on free surfaces, CAL of 5 mm on the buccal surface, absence of interproximal bone loss, slight (dental linguoversion) on the buccal surface, biofilm accumulation, protruding occlusal interference, and thin periodontal phenotype (Figure 4).

**Figure 5:**
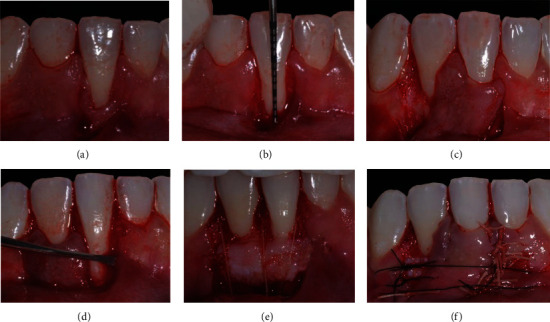
Case 2. (a) Vertical internal bevel incision extending into alveolar mucosa and external bevel incision along distal gingival margin of gingival recession (GR) defect. (b) Deepithelialized area after intrasulcular incision. (c) Flap of mix thickness. (d) Mesial papilla of tooth 41 deepithelialized and tunnel preparation. (e) Connective tissue graft (CTG) positioned and stabilized with sutures. (f) Laterally positioned flap without tension and secured with sutures on the recipient site.

Firstly, an adjustment of protrusive occlusal interference in tooth #41 was performed, as describe in case 1. Two weeks after the occlusal adjustment, periodontal surgical treatment was performed using the LPF + SCTG technique (T.M.M.; Figure 5).

### 2.4. Case 3

**Figure 6:**
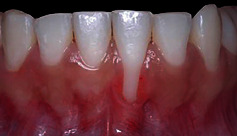
Case 3. Frontal view, gingival recession of 5 mm high by 3.5 mm wide.

The patient, a 23-year-old, Caucasian woman, systemically healthy, presented with main complaint of fear of losing tooth #31 and dentin hipersensivity, at the Department of Surgery and Integrated Clinic, São Paulo State University (Araçatuba, São Paulo, Brazil), in April 2016. The patient was a nonsmoker and had previously undergone orthodontic treatment. According to the clinical and radiographic examination, an RT1 RG was observed in the buccal surface of the left mandibular central incisor (5 mm high and 3,5 mm wide at the height of the CEJ), with a PD of 2 mm in the proximal and 2 mm on free surfaces, CAL of 7 mm on the buccal surface, absence of interproximal bone loss, biofilm accumulation, and thin periodontal phenotype (Figure 6).

**Figure 7:**
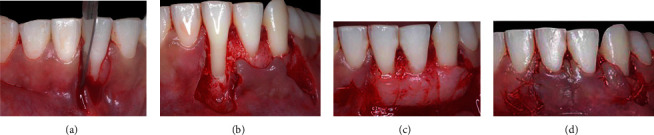
Case 3. (a) Vertical internal bevel incision extending in alveolar mucosa and an external bevel incision along distal gingival margin of gingival recession (GR) defect. (b) Flap of mix thickness. (c) CTG positioned and stabilized with sutures. (d) Laterally positioned flap without tension and secured with sutures on the recipient site.

After 21 days of the supragingival periodontal therapy, the treatment using the LPF + SCTG technique was performed in a single surgical stage (J.M.A.). In this case, the flap was extended to two adjacent teeth distal tooth with GR, so that it remained of mixed thickness on the immediately adjacent tooth (tooth #32) and split thickness on the most distal tooth (tooth #33). The CTG was extended mesiodistal enough to cover tooth #31 to #33 (decision made due to the width [3.5 mm] and height [5 mm] of the GR), to cover the exposed root surface and remain within the tunnel created under the initial mesial incision of tooth #31, to later be covered by LPF (Figure 7).

**Figure 8:**
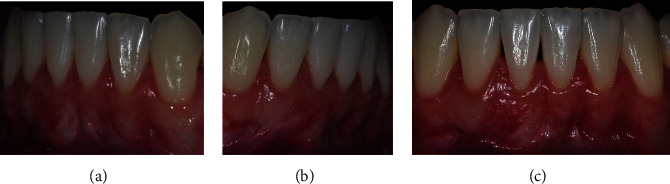
Case 1. Healing 18 months postoperatively. (a) Left side view showing complete root coverage. (b) Right side view showing maintenance of integrity of donor bed. (c) Front view showing keratinized tissue width gain and gingival tissue color very similar to that of the adjacent area.

**Figure 9:**
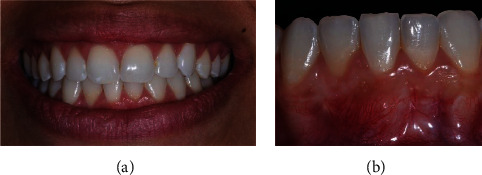
Case 2. (a) Frontal aspect of smile with visualization after healing 24 months postoperatively. (b) Front view showing complete root coverage and keratinized tissue width gain.

**Figure 10:**
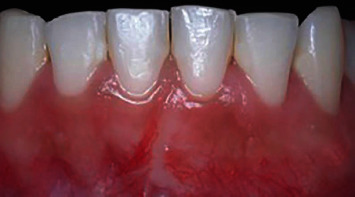
Case 3. Frontal view, healing 48 months postoperatively demonstrating complete root coverage; maintenance of integrity of the donor flap area, keratinized tissue width gain, and gingival tissue color very similar to that of the adjacent area.

Three deep GRs were surgically treated with LPF + SCTG in three different patients. The periodontal characteristics of the three clinical cases are presented in Table 1. One hundred percent of the cases treated achieved CRC. An increase in the keratinized tissue width (KTW) was observed in all the cases. The follow-up time intervals were 18, 24, and 48 months, in cases 1, 2 and 3, respectively (Figures 8–10). No postsurgical complications were reported.

## 3. Discussion

This manuscript described a modified one-stage procedure to perform the LPF + SCTG as an effective therapeutic option for the treatment of deep isolated GRs in mandibular incisors. In time intervals of 18 (case 1), 24 (case 2), and 48 months (case 3) after surgeries, CRC was obtained in all defects treated. Mean RD and MRC changed from 4.33 ± 0.58 mm at baseline to 0.0 ± 0.0 mm (*p* < 0.05) in the last postoperative period of each case. The CRC was obtained not only in Cairo RT1 defects (cases 2 and 3) but also in the Cairo RT2 GR (case 1). Mean KTW changed from 0.0 ± 0.0 mm at baseline to 5.0 ± 2.0 mm (*p* < 0.05) in the follow-up period. The donor bed of the LPF showed no postoperative GR defect in follow-up time intervals. The patients reported absence of dentin hypersensitivity when cleaning the region of GR defects and an excellent esthetic result of periodontal tissues.

The treatment of deep buccal GRs in the mandibular anterior area represents a major clinical challenge owing to several anatomical conditions [20, 21]. In this case series, the decision-making to define the flap extension depended on the recession depth (GR ≥ 5 mm) and width (GR ≥ 3.5 mm) of the GR and on the presence or absence of compromising factors, such as buccal tooth position, root prominence, proximity of vital structures (e.g., mental nerve), and deep bone dehiscence [20].

The potential of the FGG (free gingival graft) for increasing the KTW has been documented and seems to explain the increase in KTW after the two-stage CAF procedures [21, 22]. Among the free graft procedures, the bilaminar techniques have been reported to be more predictable [23] and to provide more esthetic results [24] than the FGG. The surgical technique (LPF + SCTG) presented in this case reports has been shown to be effective in increasing KTW and root coverage in only one surgical procedure [8], thereby minimizing morbidity among surgical patients.

In a randomized controlled clinical study, [23] evaluated the treatment of isolated Miller Class I and II GR on mandibular incisors. Treatment was performed by means of CAF + SCTG with or without removal of labial submucosal tissue (LST). The results showed predictable recession coverage, while the additional removal of LST yielded a tension-free flap, resulting in less graft exposure and statistically significantly better CRC (48% vs 88%). These results were difficult to compare directly with those obtained in the present case series, which also included one case of RT2 GR, and the donor area of the flap was lateral to the GR defect. Nevertheless, both studies clearly pointed out the pivotal role of tension-free mobilization of the flap to obtain predictable CRC.

The results of the KTW gain obtained with LPF + SCTG (from 0.0 ± 0.0 mm at baseline to 5.0 ± 2.0 mm at follow up times) were similar to those found by César Neto et al. [20] who reported the results of root coverage in maxillary and mandible isolated Miller Class II or III deep GRs (≥ 5 mm), based on the decision-making algorithm. Similarly, Sculean and Allen [24] obtained KTW values of 1.41 ± 1.00 mm at baseline to 4.14 ± 1.67 mm at 12 months, generating a gain of 2.75 ± 1.52 mm, in a series of 24 patients treated by means of a novel surgical technique (the laterally closed tunnel, LCT) associated with CTG, specifically designed for Miller Class I, II, or III deep isolated mandibular GR (≥ 4 mm).

The use of the split thickness flap in the most distal portion of the flap as described in the surgical technique prevented bone exposure and possible gingival recession in that area [26]. The performance of internal and external bevels characterizes the inversion of bevels concerning Parkinson et al. [13], which used an external bevel in the donor area and an internal bevel in the flap positioned laterally. In the present technique (LPF + SCTG), it is the opposite of this (Figure 1(a)). As the flap was positioned laterally, it is important to report that the flap bevel inversions [14, 15] were used to allow better adaptation at the time of suturing, in order to avoid overlapping the flap in the receiving area and thereby reducing visibility of the incision line perception after scarring.

Zucchelli et al. [9] evaluated the effectiveness concerning root coverage of a modified surgical approach of the laterally moved flap procedure with a submarginal incision and mix-thickness flap in the donor site, for the treatment of the isolated type of recession defects (Miller Class I or II). Specific features of the KT lateral to the defects were considered for indication: lateral keratinized tissue width (KTW donor) at least 6 mm greater than the width of the recession measured at the level of the CEJ and lateral keratinized tissue height (KTH donor) at least 2 mm greater than the buccal probing depth (PD) of the adjacent tooth/teeth (PD donor). Unlike the technique described by Zucchelli et al. [9], the present LPF + SCTG technique considers adding a tunneled CTG in the GR mesial and distally extended to protect and change the gingival phenotype to thicker areas adjacent to the GR defect. In the anterior mandible, a thin gingival phenotype is often found. It can be difficult to move the flap in the coronal direction due to muscle insertions and shallow vestibule [23]. In this context, the LPF + SCTG technique considers that the preservation of the entire band of KT adjacent to GR in the flap minimizes this need to move the flap also in the coronal direction to cover the GR defect.

A minimum amount of the attached keratinized tissue (KT) (≥ 1 mm) is required to prevent significant apical displacement of the gingival margin in the long-term [27]. In addition, it was shown that gingival thickness > 1.2 mm at the level of the keratinized mucosa was a positive predictor of CRC [24]. In the presence of a thin gingival phenotype (< 1 mm GT), bilaminar procedures consisting of a LCT or a LPF in combination with an autogenous graft are recommended [15, 24, 26]. In the LPF + SCTG technique described, the graft is inserted into the created tunnel and extended distally to protect the donor area of the flap, even if a small portion of it is exposed in that area [27]. In this clinical setting, the gingival phenotype of the areas adjacent to the GR defect were modified by obtaining greater thickness and KTW gain, which may favor the stability of long-term root coverage results [15].

LPF + SCTG and CAF + SCTG are commonly used surgical approaches to treat deep localized GR in mandibular incisors. However, in the presence of a shallow vestibule, Cairo RT3 GRs or lack of the keratinized tissue (minimum of 2 mm) adjacent to GR results using these approaches has clear limitations [28]. These clinical situations may represent the main indications for the two-step surgical approach with flattening of the root surface and later LCT [28]. In this two-step approach, flattening of the root surface creates a new emergence in order to treat anterior mandibular localized GRs in the prominent teeth. This approach will provide more space for the graft, increase the thickness of the gingival margin, and provide extra soft tissue at the margins of the GR [28].

The mandibular incisors of the cases described were relatively well positioned in the bone envelope. Although we did not perform the cone-bean computed tomography to observe the buccal-lingual inclination of the roots and the occurrence of bone dehiscence in the teeth adjacent to the GR defect, this is considered an important complementary exam for making the treatment decision about whether to perform orthodontic treatment or concomitant odontoplasty to the surgical technique of root coverage [28].

Despite the excellent results obtained with the LPF + SCTG technique in the cases reported, these findings revealed the importance of well-designed randomized clinical studies that compare the effectiveness of different surgical techniques for the treatment of deep isolated GRs in the mandibular arch.

The present results indicated that the LPF-SCTG with the modifications presented is a predictable approach for the treatment of deep isolated RT1 and RT2 GRs in mandibular incisors that are well positioned in the bone envelope with the presence of KTW adjacent to GR and adequate vestibule depth in the donor area.

## Figures and Tables

**Table 1:** Clinical characteristics of the three clinical cases, means (±SD), minimum (min), and maximum (max) of clinical parameters at baseline and follow-up.

Variable	Time point	Mean ± SD	Min	Max	*p* value Paired *t*-test
PD (mm)	Baseline	2.06 ± 0.42	1.67	2.50	>0.05
	Follow-up	1.83 ± 0.29	1.67	2.17	

PD-3b (mm)	Baseline	2.11 ± 0.51	1.67	2.67	>0.05
	Follow-up	1.89 ± 0.38	1.67	2.33	

PD-1b (mm)	Baseline	1.67 ± 0.58	1.00	2.00	≤0.001
	Follow-up	1.00 ± 0.00	1.00	1.00	

CAL (mm)	Baseline	1.56 ± 0.98	0.83	2.67	0.017
	Follow-up	0.44 ± 0.77	0.00	1.33	

CAL-3b (mm)	Baseline	2.44 ± 0.84	1.67	3.33	0.00
	Follow-up	0.44 ± 0.77	0.00	1.33	

CAL-1b (mm)	Baseline	6.00 ± 1.00	5.00	7.00	0.009
	Follow-up	0.00 ± 0.00	0.00	0.00	

KTW (mm)	Baseline	0.00 ± 0.00	0.00	0.00	0.049
	Follow-up	5.00 ± 2.00	3.00	7.00	

RD (mm)	Baseline	4.33 ± 0.58	4.00	5.00	0.005
	Follow-up	0.00 ± 0.00	0.00	0.00	

MRC (mm)	Follow-up	4.33 ± 0.58	4.00	5.00	—
CRC (100%)	Follow-up	Yes	Yes	Yes	—

Abreviations: CAL, Clinical Attachment Level (6 sites); CAL-3b, Clinical Attachment Level (3 buccal sites); CAL-1b, Clinical Attachment Level (1 buccal site); CRC, Complete Root Coverage; KTW, Keratinized Tissue Width; mm, millimeters; MRC: mean root coverage; PD: probing depth (6 sites); PD-3b: probing depth (3 buccal sites); PD-1b: probing depth (1 buccal site); RD: recession depth; SD: standard deviation. The significance of differences over time was assessed by paired *t*-test (*p* < 0.05).

## Data Availability

Periodontal clinical data from each clinical case was used for this manuscript.
